# Animals for the Deceased: Zooarchaeological Analysis of the Bronze Age in the Castillejo del Bonete Site (Terrinches, Ciudad Real, Spain)

**DOI:** 10.3390/ani15050680

**Published:** 2025-02-26

**Authors:** María Ángeles Galindo-Pellicena, Amalia Pérez-Romero, Andrea Gómez-Felipe, Marta Romero-Ruiz, Raquel Blázquez-Orta, Silvia Andreu-Alarcón, Luis Benítez de Lugo Enrich

**Affiliations:** 1 Centro Mixto UCM-ISCIII de Evolución y Comportamiento Humanos, C/Monforte de Lemos 5, Pabellón 14, 28029 Madrid, Spain; 2 Laboratorio Evolución Humana, Universidad de Burgos, C/Misael Bañuelos s/n, 09001 Burgos, Spain; apromero14@gmail.com; 3 Escuela de Doctorado, Universidad de Alcalá de Henares, Calle Libreros 21, 28801 Alcalá de Henares, Spain; andrea.gomezfelipe@edu.uah.es; 4 Independent Researcher, 28040 Madrid, Spain; marome30@ucm.es; 5 Research Goup: Ecosistemas Cuaternarios, Universidad Complutense de Madrid, 28040 Madrid, Spain; rborta@ucm.es; 6 Departamento de Geodinámica, Estratigrafía y Paleontología, Facultad Ciencias Geológicas, Universidad Complutense de Madrid, 28040 Madrid, Spain; 7 Independent Researcher, 28792 Madrid, Spain; silviaandreua@gmail.com; 8 Departamento de Prehistoria, Historia Antigua y Arqueología, Facultad de Geografía e Historia, Universidad Complutense de Madrid, 28040 Madrid, Spain

**Keywords:** bronze age, Castilla La Mancha, Spain, zooarchaeology, taphonomy

## Abstract

The 2978 faunal bone remains recovered from the Bronze Age levels at the Castillejo del Bonete site between 2005 and 2019 were analyzed in this study. In the main structure (Great Tumulus 1, Tumulus 2, and Tomb 5), the faunal bone remains were identified and interpreted as offerings in a funerary context. In Enclosure 4, a large building possibly dedicated to the celebration of wakes and rituals—including the eating of food and drinking—in honor of the deceased, the anthropic modifications on the bone surfaces indicate their possible consumption. The cave is constituted by four galleries with inhumations (in Galleries 2, 3, and 4) and pithoi (in Gallery 4). Wild and domestic animals were identified in the cavity, and the caprine (likely sheep) is the best represented taxa in the entire cave. The almost complete skeletal representation; the predominance of fetal, neonate, and juvenile individuals; the absence of anthropic modifications related to consumption, in addition to the bone industry made of caprine bones (such as an eye idol and anthropomorphic artefacts), suggest that caprines were used as offerings to the deceased that accompany them. Postdepositional taphonomic alterations, the animals not having been found in anatomical connection, and the refits of the bones between different stratigraphical units indicate the repeated use of the cave during the Bronze Age.

## Simple Summary

This paper presents the zooarchaeological and taphonomic analyses of the bone remains recovered during fourteen field campaigns. The Castillejo del Bonete site constitutes a massive tumular and astronomic monument from the Bronze Age located on the southern edge of the Southern Plateau in the village of Terrinches, Ciudad Real, Spain. The faunal remains study reveals the importance of caprines as offerings to the deceased with symbolic character and occasional rituals, which was suggested by the sporadic consumption identified in some of the analyzed bones.

## 1. Introduction

The animals that accompany a human population during Prehistory provide a reflection of their way of life and illustrate their relevance and the role they played at that time. Faunal bone remains have been recovered from funerary contexts at archaeological sites from the Chalcolithic and Bronze Age in the Iberian Peninsula (the Chalcolithic in the El Portalón site, Atapuerca, Burgos [1]; the Early Bronze Age in Can Roqueta II, Sabadell [2]; the Bronze Age in Pista de Motos, Villaverde, Madrid [3]; and the Chalcolithic and Bronze Age in the Aldovea site (Torrejón de Ardoz, Madrid) [4,5]). These animals were deposited close to the human bodies found there, and they have been interpreted as offerings or the results of feasting before, during, or after a funeral [6]. The animals used as offerings can have different meanings: focus on the gods of the underworld to ensure the well-being of the deceased; focus on the deceased: offerings like food for the afterlife or as psychopomps that accompany the deceased [7,8,9,10,11,12]. The offerings of animals in tombs or burials suggest the reflection of the belongings of the deceased and, therefore, illustrate differentiations between the individuals that accompanied them [2]. The animals also could be the result of feasting related to a funeral.

The analysis of the representation of anatomical elements and human processing marks can infer the different uses of the animals in funerary contexts [13]. There are some criteria defined by [2] to distinguish funerary contexts with faunal bone remains that are interpreted as offerings of a symbolic character from faunal bone remains related to feasts.

The faunal remains associated with a symbolic nature can have the following characteristics:

- More than one animal in anatomical connection can suggest the intentionality of the whole and eliminates doubt regarding whether the deposit is coincidental or intentional for hygienic purposes.
- Majority selection of important species in production (including domestic ones).
- Structured arrangement of the skeletal parts and of whole animals in space.
- Appearance of anatomical parts of high content in flesh, and therefore, characteristic of the typical diet, with low fracture rates. These can be interpreted as meat offerings to the deceased, but no significant volume of remains has been documented.
- Preference regarding laterality (which side has an important symbolic meaning in the ritual offerings).
- Abundance of one sex and of ages or very young or adult (female specimens associated with women, while the male is associated with men).
- Association with or proximity to human remains as well as a relationship with archaeological material of various kinds (vessels, mills, remains, charred vegetables, etc.).

Faunal bone remains derived from funeral banquets can have the following characteristics:

- Animals are not usually documented in anatomical connection. High frequency of fractures and cut marks related to the lack of flesh.
- Majority selection of important species in production (including domestic ones).
- There is no evidence of a structured arrangement of skeletal remains in space.
- Appearance of a considerable volume of remains among which the parts are predominantly anatomical, of high meat content, and therefore, important to the diet.
- There is no preference for laterality.
- Abundance of remains that are inferred to be adult ages.
- Association or proximity to human remains

In this manuscript, the zooarchaeological and taphonomic analyses have been conducted on the remains from two different areas in the Castillejo del Bonete site (tumular structure and cave).

There were two main objectives:

- To infer the use of the animals;
- To try to infer the nature (symbolic/feast) of the animal deposits from the human population.

**The site**

**Figure 1:**
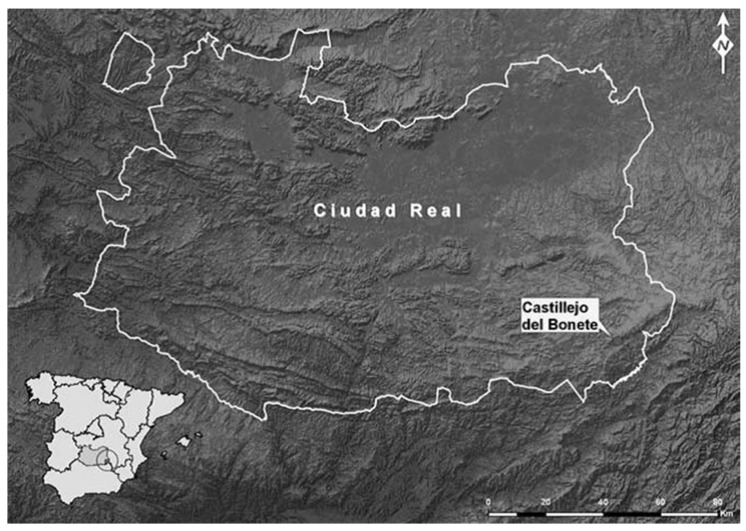
Location of the archaeological site of Castillejo del Bonete (Terrinches, Ciudad Real). Taken from [17].

Castillejo del Bonete is a site located in Terrinches, Campo de Montiel, in Ciudad Real (Spain) (Figure 1). It is a Bronze Age archaeological site located on the southern edge of the Southern Plateau to the north of the Sierra Morena Mountain range and within the Guadalquivir River basin. It is a monumental tumular structure with corridors and tumuli built over a cave. This site’s record consists of funerary remains, votive deposits, lithic and bone industry, pottery, and bone remains [14,15].

At the base of this mountain range, there is a natural corridor between the Meseta, Alta Andalucía and Levante: a passage which would become known as the Via de los Vasos de Vicarello (four silver vessels that were deposited as votive offerings in the thermal waters of Lake Bracciano, Acquae Apollinares, near Rome) many centuries later. Its character as a territorial marker seems clear [16].

**Figure 2:**
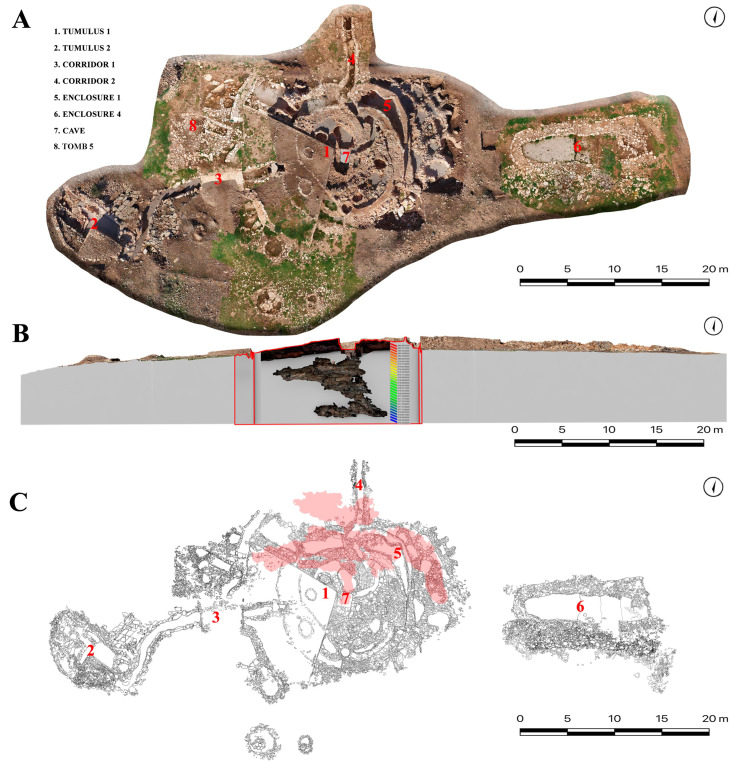
Map (**A**) and sectioning (**B**) of the tumular structure and cave at the Castillejo del Bonete site. The numbers in the column represent heights measured in relation to sea level. Lineal design of the tumular structure (**C**). For (**A**) and (**C**): 1. Tumulus 1; 2. Tumulus 2; 3. Corridor 1; 4. Corridor 2; 5. Enclosure 1; 6. Enclosure 4; 7. Cave; 8. Tomb 5. Picture modified from [19].

The Castillejo del Bonete site is a monumental complex that is formed by its biggest building, called Great Tumulus 1 (1 in Figure 2A), with “deposits” of archeological and faunal remains in Tumulus 2 (2 in Figure 2A), Tomb 5 (8 in Figure 2A), Enclosure 4 (6 in Figure 2A), and Corridors 1 and 2 (3 and 4 in Figure 2A). Great Tumulus 1 is located in a natural cave (Sima: 7 in Figure 2A) that was used as a tomb, which has schematic post-Palaeolithic cave art depicted inside [18].

Great Tumulus 1 measures 44.2 m in diameter from north to south (80 m perimeter). Tumulus 2 is a secondary tumular structure connected to the main tumulus by a winding corridor (Corridor I), which measures 12.1 m (32.6 m perimeter). Great Tumulus 1 is divided into more than thirty pits or negative structures, some of which were found empty, while others contained archaeological materials from different kinds of sites, which were called “deposits”. These structures have been interpreted as sites where offerings of a ritualistic character were made similar to those documented in other sites ([15,19]). The site comprises five corridors of varying types. Some corridors allow the rays of the sun to shine into the interior of the monument at sunrise and sunset for many weeks, thus leading to its interpretation as an astronomical monument. Tomb 5 is located close to Tumulus 1. Enclosure 4 is a building located rather far from the funerary tumulus. No burials have been documented in this monument. Thus, its use was not funerary but rather for gatherings of the living in honor of the deceased ([15,19]). The funerary tumulus, Great Tumulus 1, was built above a sepulchral grave cave. The cave is composed of four galleries (1 in Figure 2B). Every gallery is divided into different sectors. In Gallery 3, five inhumations were identified, and in Gallery 4, two pithoi were recovered. The stratigraphy of these galleries indicates successive movements in the cavities, which indicate secondary positioning of the bone elements.

The complete taxonomic and taphonomic analyses of the faunal bone remains recovered from the Castillejo del Bonete site are provided for the first time in this manuscript.

## 2. Materials and Methods

A total of 2978 faunal remains from the Bronze Age at the Castillejo del Bonete site were recovered: 425 faunal bone remains were identified in the tumular structures (194 in the different “deposits”, 78 in Tumulus 2, 24 in Tomb 5, and 129 in Enclosure 4). In addition, 2553 faunal bone remains found in the cave were analyzed: 631 in Gallery 2, 672 in Gallery 3, and 1250 in Gallery 4.

The anatomical and taxonomic identification of the faunal remains from the Castillejo del Bonete site was carried out using atlases of animal anatomy [20,21,22] and the collection of comparative anatomy at Centro Mixto UCM-ISCIII de Evolución y Comportamiento Humanos in Madrid. In the cases in which taxonomic and/or anatomical identification was not possible, the animals were classified into three size categories, depending on the weight of the different species, according to the classification of [23], including suids: (a) large size: cows, horses and large cervids; (b) medium size: small ruminants, pigs, boars, wolves and dogs; and (c) small size: small dogs, cats, hares and rabbits.

Discrimination between sheep (*Ovis aries*) and goats (*Capra hircus*) was determined based on the diagnostic characteristics following [24,25,26] and publications on the dental differences between the two taxa in accordance with [27,28,29,30]. The metric method proposed by [31] for distinguishing *Ovis* and *Capra* based on the distal part of the metacarpals using DEM (medial trochlea depth) and WCM (medial condyle width) was so applied. The remains that could not be identified were assigned the generic denomination “caprine”. The *Ovis aries* and *Capra hircus* metrical data from the Iron, Roman and Medieval periods in Santarém [32], in addition to the reference collection of the *Ovis* and *Capra* population from the archaeological laboratory at the University of Sheffield (LASH) and the Museum of Natural History MNHN (París), taken by MGP, one of this study’s authors, were used for the discrimination.

The bones were quantified using the following criteria: Number of Identified Specimens (NISP), Minimal Number of Elements (MNE), Minimal Animal Units (MAU), standardized %MAU and Minimal Number of Individuals (MNI) [33]. The MAU were calculated by standardizing the MNE values according to the number of times an anatomical element occurs in the skeleton in order to mitigate the overrepresentation of ribs and vertebrae. We used the following anatomical elements for this quantification: cranium (CRN); hemimandible (HMN); atlas (ATL); axis (AX); cervical vertebra 3e7 (CEV 3e7); thoracic vertebra (TV); indeterminate vertebra (IV); rib (RB); sternum (ST); lumbar vertebra (LMV); sacrum (SA). caudal vertebra (CAV); scapula (SC); humerus (HM); radius (RD); ulna (UL); unciform (UCF); semilunar (SEMIL); scaphoid (SCAP); metacarpal (MC); innominate (IM); femur (FM); patella (PT); tibia (TA); talus (AS); calcaneus (CL); scapho-cuboid (SCAP-CUB); metatarsal (MT); indeterminate metapodial (IMP); first phalange (PHF); second phalange (PHS); third phalange (PHT).

The NISP by SU was included in tables, and an explanation of the faunal remains was included in the text for the cases in which the NISP was greater than 50, which were considered relevant. A detailed zooarchaeological analysis was only possible for caprines. In the cases where the caprine NISP exceeded 100 bone remains, the skeletal parts representation by elements was carried out.

The age at death was established by taking into account the eruption and dental wear and stages of bone fusion. In caprines, the wear stage for all P4s, dP4s, and molars, both isolated and in mandibles, was recorded following [34,35]. Those mandibles with at least two teeth in the dP4/P4-M3 row were assigned to mandibular wear stages proposed by [35]. The methodology implemented by [36] for mandibles and [37] for maxilla was used for suids. The fusion of the bones was established following [22].

Caprines were classified by their age at death according to the criteria established by [38], who distinguish fetuses (0 to 2 months) from neonates (less than 3 months), and according to [39] for juveniles (from 5/6 to 18 m), subadults (from 18 to 48 m), adults (48–120 m), and (>120 m) senile.

The funerary context in the galleries was not found in primary position, and therefore the animals and human skeleton were not found in anatomical connection. Nevertheless, this does not mean that they were not in anatomical connection at the time of placement. Skeletal representation is essential for inferring the way the animals were placed close to the human in the burial or inhumation. Therefore, the anatomical elements were associated by taking into account the laterality, dimensions, and fusion stages of the skeletal elements. After carrying out the associations and determining the integrity of the carcasses, the AEAs (anatomical element associations) were classified into three different categories: complete skeletons (C), incomplete skeletons (partial: P), and isolated (isol) remains (a category that includes limb bones deposited individually or linked to other sections as well as trunk bones—ribs, shoulder blades and pelvis—in addition to skulls and jaws), according to [2,40].

All the faunal bone remains were considered in the taphonomic analysis. For the microscopic study, a DINO-LITE digital microscope of AnMo Electronics Corporation, designed in Taiwan (the manufacturer was from The Netherlands, and the equipment was sourced from Barcelona, Spain), and Dinocapture 2.0 sofware were used. The taphonomic analysis focused on human modifications: cut marks, bone breakage, and heat modifications [41,42,43]. The location of cut marks was recorded—especially the muscle insertion areas or if they were found on tendons or ligaments—since these can be used as criteria for distinguishing different butchery activities [44,45,46]. In order to study the burned bones, we followed the stages defined by [47].

Postdepositional surface modifications were also recorded, including carnivore and rodent gnawing, root etching, bone weathering stages, etc. [48], which were grouped under the generic term “biochemical activities”.

### Dating of the Site

At the Castillejo del Bonete site, seven radiocarbon dates were obtained from different stratigraphic layers (Table 1). These results dated the site to a Bronze Age chronology.

## 3. Results

### 3.1. Taxonomical Identification

*Ovis aries*/*Capra hircus*

**Figure 3:**
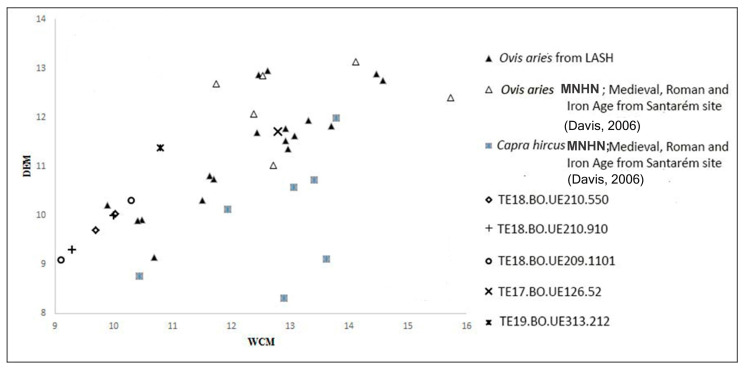
*Scatter plot* (DEM: medial trochlea depth; WCM: medial condyle width) of *Ovis aries* from LASH, MNHN and metrical data from Santarém site taken to [32] and *Capra hircus* from MNHN and the Santarém site [32] and caprine metrical data from the Castillejo del Bonete site.

A metrical analysis was performed on the distal part of caprine metacarpals in order to distinguish between sheep and goats. Figure 3 compares the measurements of the caprines from Castillejo del Bonete to those of *Ovis aries* and *Capra hircus* populations from different collections, including both recent and specimens taken from prehistoric sites. The distal parts of five metacarpals were measured, and these five metacarpals fall into the range of metrical variation in the *Ovis aries*; thus, the metacarpals are believed to belong to *Ovis aries* (Table 2 and Figure 3).

### 3.2. Zooarchaeological Analysis

The map depicts the different contexts where the faunal analysis was carried out from east to west (Figure 2):

The outer tumulus structure is analyzed in the different burials.

**TUMULAR STRUCTURE**

The faunal remains classified in the “deposits” defined in Great Tumulus 1 were analyzed:

“Deposit 5” includes SU30 (SU: stratigraphical unit), where an upper m^1^/m^2^ from a caprine and a mandible with m_2_ and m_3_ erupting were recovered, belonging to a subadult *Sus* sp.

“Deposit 10” includes SU75, where a mandible with m_2_ and m_3_ erupting that belonged to a subadult *Sus* sp. was recovered in addition to a caprine incisive.

“Deposit 12” includes SU60 where an upper molariform belonging to a caprine and a *Oryctolagus cuniculus* talus were identified.

“Deposit 14” includes SU37, where 17 faunal bone remains were identified, mostly caprines (10: 3), belonging to a minimum number of three individuals (MNI) (seven dental elements): two adults (stages G and H from [35]) and one juvenile. Seven bone remains belonging to medium-sized animals were also identified.

“Deposit 17” includes SU86, where seven faunal bone remains were identified, which mostly belonged to caprines (5: MNI = 2) and were attributed to two individuals (stage C and stage E of [35]). Additionally, two dental elements were identified, belonging to immature *Sus* sp.

“Deposit 19” includes SU99, where one caprine molar was identified.

“Deposit 33” includes SU154, where 11 faunal remains were identified: caprines (3:1), *Bos taurus* (2:1), *Cervus elaphus* (2:1), and medium-sized animals (4).

“Deposit 36” includes SU 174 with 32 faunal bone remains: caprines (7:1), large-sized animals (15), and small-sized animals (1).

“Deposit 37” includes SU233 and 234. In SU 233, 26 faunal bone remains were identified: caprines (3:1), *Sus* sp. (2:1), *Bos taurus* (1:1), large-sized animals (1:1), medium-sized animals (16), and small-sized animals (3). In SU 234, four faunal bone remains were identified: caprines (3:1) and *Sus* sp. (1:1).

“Deposit 43” includes SU337 and 339. In SU 337, 23 faunal bone remains were identified: caprines (5:1), *Sus* sp. (2:1), *Oryctolagus cuniculus* (4:1), large-sized animals (1:1), medium-sized animals (10), and small-sized animals (1). In SU 339, 13 faunal bone remains were identified: caprines (3:1); *Sus* sp. (2:1), *Oryctolagus cuniculus* (5:1), *Bos taurus* (1.1), and medium-sized animals (2:1).

“Deposit 44” includes SU 260, which yielded 35 faunal bone remains: caprines (19:1), which belonged to an almost complete caprine skeleton, *Bos taurus* (1:1), *Sus* sp. (3:1), *Oryctolagus cuniculus* (2:1), large-sized animals (2:1), medium-sized animals (7), and small-sized animals (1).

“Deposit 46” includes SU 340, where 19 faunal bone remains were recovered: caprines (4:1), *Sus* sp. (1.1), *Oryctolagus cuniculus* (3:1), large-sized animals (4), medium-sized animals (5), and small-sized animals (2).

**Tumulus 2** (2 in Figure 2A)

SU194

A total of 78 faunal bone remains were identified, 32 of which belonged to a minimum number of one caprine, with an almost complete (C) skeletal representation, and age at death of less than 36 months (indicated by an unfused metatarsal) (32:1), *Sus* sp. (5:1), *Bos taurus* (1:1), *Oryctolagus cuniculus* (3:1), indeterminate bird (2), middle-sized animals (33), and small-sized animals (2).

**Tomb 5** (8 in the Figure 2A)

Tomb 5 includes SU257, where a total of 24 faunal bone remains were identified: caprines (1:1), *Sus* sp. (1:1), Leporidae indet. (1:1), *Lepus granatensis* (2:1), *Oryctolagus cuniculus* (2), and medium-sized animals (17).

**Enclosure 4** (6 in the Figure 2A)

A total of 129 faunal bone remains were identified in Enclosure 4.

SU 184. A total of 113 faunal bone remains were identified, nine of which were classified as unidentified. The best represented taxa are caprines (29:1), which were followed by *Sus* sp. (12:1), belonging to a juvenile individual (mandible + dp4), *Bos taurus* (5:1), *Oryctolagus cuniculus* (1), large-sized animals (18), medium-sized animals (45), and small-sized animals (3).

SU 1 covers most of the site. Sixteen bone remains were included in this SU located in Enclosure 4: nine belonging to caprines and seven belonging to *Bos taurus* (two fragments from the same phalanx and five teeth belonging to the same individual). Biochemical modifications and pits were observed on some of the remains.

**CAVE** (7 in the Figure 2A)

**Gallery 2**

A total of 631 faunal bone remains were identified in Gallery 2 (Table 3 and Table 4). 

Gallery 2 is composed of different stratigraphic units that are displayed in Table 4. Caprines are the best represented taxa in most cases (see Supplementary Material for the detailed data). SU 217, 229, and 26013 contain 39, 29, and 56 bone remains, respectively, which are all attributed to caprines (50.6%, 18.2% and 22.3%).

**Figure 4:**
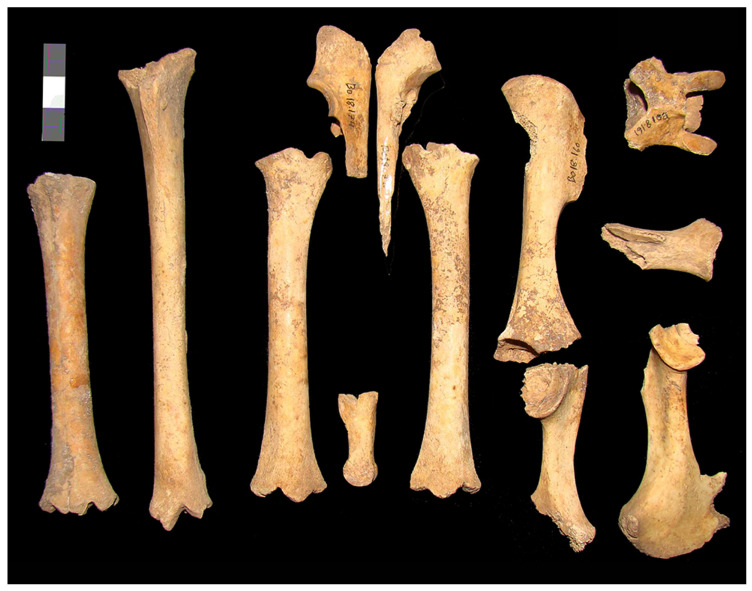
Association of complete anatomical elements belonging to the same caprine individual from SU217 in Gallery 2.

**Figure 5:**
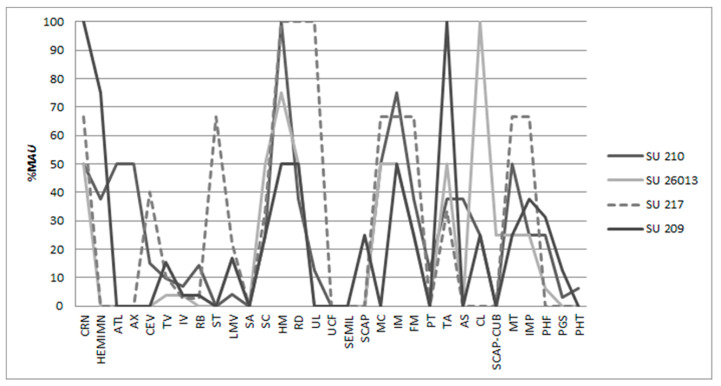
Skeletal part representation by caprine elements (%MAU) from SUs 209, 210, 217 and 26013. For abbreviations, see Methodology.

With respect to the caprine skeletal representation, while the animals were not found in anatomical connection, the associations of the complete skeletal elements are notable. In some cases, it was possible to nearly reconstruct the caprine skeletons (Figure 4). The relative abundance of caprine elements (%MAU) in SU 217 and 26013 is graphically observed in Figure 5. In SU 217, the %MAU of caprine forelimbs and hind limbs is 75 and 40, respectively, while 100 is the %MAU for cranial remains, 12.5 for axial, and 4 for phalanges. In SU 26013, the %MAU of caprine forelimbs and hind limbs suggest a similar representation (around 30%); the %MAU of cranial and mandibles is 100 and 75, respectively. Furthermore, the %MAU of axial postcranial elements and phalanges is around 5%, and thus all anatomical elements are represented.

Moreover, two complete tibias of *Lepus granatensis* were associated with the same individual, though they were found in different stratigraphical units (TE18.BO.UE217. 713 and TE18.BO.UE229.787). The two *Cervus elaphus* metatarsal bone fragments (TE18.BO.UE230.770+921 with TE18.BO.UE217.205) recovered from different stratigraphical units were refitted.

A minimum number of 16 caprine individuals was calculated for Gallery 2. The age at death for 11 of them was determined: two fetals, one neonate, four juveniles, and four adults; therefore, 63% of the individuals were slaughtered before 48 months of age (F, N, J, SA). In terms of the integrity of the skeletons, one was recovered almost complete, eight in partial parts, and two in isolated parts (Table 4).

**Gallery 3**

A total of 672 faunal bone remains were identified in Gallery 3 (Table 5 and Table 6 for MNI of caprines). 

Gallery 3 was divided into different sectors, in which five inhumations were identified. In Inhumations 1,2, 4, and 5, caprines constitute the best represented animal (see Supplementary Materials for the detailed data)

A minimum number of 32 caprine individuals were calculated in Gallery 3 (Table 6). The age at death for 22 of them could be determined: three fetals, four neonates, two juveniles, six subadults, and seven adults, and thus 68.2% of the individuals were slaughtered before reaching 48 months of age (F, N, J, SA). Regarding the integrity of the skeletons, these 22 individuals (100%) were recovered in partial parts.

**Gallery 4** (SU 156, 202, 206: pithoi 2, 209, 210, 211, 212, 224, 225, 237, 244: pithoi 1)

A total of 1250 faunal bone remains were recovered: 985 were classified as identified remains (approximately 80%) (Table 7), and 265 bone remains were included in the indeterminable remains category (20%).

Gallery 4 is composed of different stratigraphical units (see Table 7 for NISP and Table 8 for MNI for caprines). The stratigraphic units that include more than 30 bone remains were described (SU156, 202, 206, 225) and included in Supplementary Materials.

The following are the most relevant units:

SU 209

A total of 135 faunal bone remains were recovered from SU 209: caprines (87:4), *Bos taurus* (1:1), *Sus domesticus* (2:1), and *Oryctolagus cuniculus* (8). Four bone remains belong to large-sized animals, 32 belong to medium-sized animals, and one belongs to small-sized animals.

A minimum number of four ovicaprine individuals were recovered from this unit: one neonate represented by an almost complete skeletal carcass, another two neonates (identified by three left dp_3_) represented by a partial carcass, and one adult with a partial skeleton. Cut marks were identified on three bone remains (2.22%).

The relative abundance of caprine elements (%MAU) in SU 209 is graphically observed in Figure 5. The anatomical elements representing about 20% of the MAU include forelimbs (18.75%) and phalanges (17.86%), while the anatomical elements that surpass 20% of the MAU belong to cranial segments.

SU 210

A total of 627 bone remains were recovered, 232 of which belong to a minimum number of seven caprine individuals (humerus): one fetal, three neonates and three adults. Regarding the skeletal representation and possible refits, two individuals could be almost completely represented: *Bos taurus* (26:1), *Canis familiaris* (3:1), *Oryctolagus cuniculus* (20:2), *Sus* sp. (18:1), *Cervus elaphus* (1:1), Carnivora indet. (6), Leporidae indet. (3), a large-sized mammal (1), medium-sized mammals (262), and small-sized mammals (23).

The relative abundance of caprine elements (%MAU) in SU 210 is graphically observed in Figure 5. The anatomical elements exceeding 50% of representation of (%MAU) are cranial and mandible elements, forelimbs, and hind limbs (100:75: 56.25: 63.33), while axial and phalanges exceed 20% (21.88: 26.79).

Sector 4.6

SU 244 (pithoi 1)

A total of 11 faunal bone remains found in pithoi 1 were identified; caprines are the best represented taxa (5:1) with one immature individual, followed by *Oryctolagus cuniculus* (2:1), *Bos taurus* (1:1), *Sus* sp. (1:1), and *Cervus elaphus* (1:1).

A minimum number of 16 caprine individuals was calculated in Gallery 4. The age at death for 15 of them could be determined: two fetals, five neonates, five juveniles, three adults, thus indicating that 80% of the individuals were slaughtered before reaching 48 months of age (F, N, J, SA). Regarding the integrity of the skeletons, there is one almost complete individual, 11 individuals were recovered in partial parts, and two were recovered in isolated parts.

### 3.3. Taphonomic Analysis

**TUMULAR STRUCTURE**

**Great Tumulus 1**

“Deposit 14” includes SU37. Two bone remains show evidence of burning (stage 3, according to [47] on the third phalanx from a caprine, and stage 4 on a long bone from a medium-sized animal).

“Deposit 37” includes SU 234, where four faunal bone remains were identified, two of which show evidence of burning (stage 1 and 4) on the surface of indeterminate bones from a medium-sized animal.

**Figure 6:**
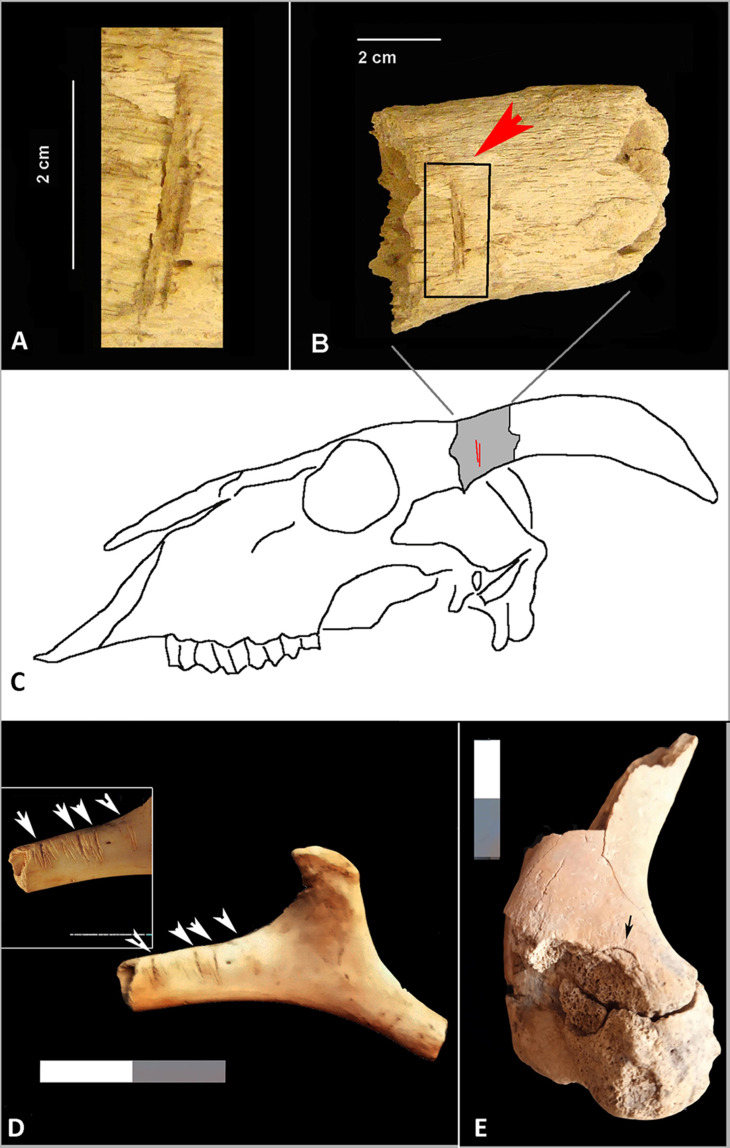
Anthropic modifications on faunal bone surfaces from the Castillejo del Bonete site. (**A**–**C**): Chop marks (red arrow) on a caprine horn core recovered from Inhumation 1 in Gallery 3; (**D**): caprine hyoid bone with slicing marks (white arrow) recovered from Gallery 4; (**E**): refit of suid humerus and impact point of percussion (black arrow) recovered from “deposit 44” in Great Tumulus 1.

“Deposit 44” includes SU 260, from which 35 faunal bone remains were analyzed. Two parts of a suid humerus were refitted, and an impact point is observed on the medial distal part (Figure 6E)

**Tumulus 2**

One caprine metatarsal shows evidence of burning, corresponding to stage 3 (fully carbonized: completely black), following the burning damage categories defined by [47].

**Enclosure 4**

Inside Enclosure 4, 120 faunal bone remains are fragments (98%); 70 bone remains (57.4%) show biochemical modifications; 31 bone remains (25.40%) show evidence of burning (stage 2 and 3 according to [47] and pits were observed on five bone remains (4.09%) in SU184.

In SU 1, included in Enclosure 4, 16 bone remains were identified. Biochemical modifications and pits were observed on some of the remains.

**CAVE**

**Gallery 2**

A total of 631 faunal bone remains were identified in the funerary context of Gallery 2. Four bone remains from the Bronze Age (0.63%) found in Gallery 2 display butchery evidence, such as cut marks. Three slices were identified on two long bones and one vertebra of a medium-sized animal’s bones and one chop mark on an *Oryctolagus cuniculus* bone, while two bone remains show evidence of burning. Following the burning damage categories as defined by [47], there is a long bone from a small-sized animal corresponding to stage 1 (less than half the surface is carbonized) and another long bone from a small animal corresponding to stage 3 (fully carbonized: completely black).

**Figure 7:**
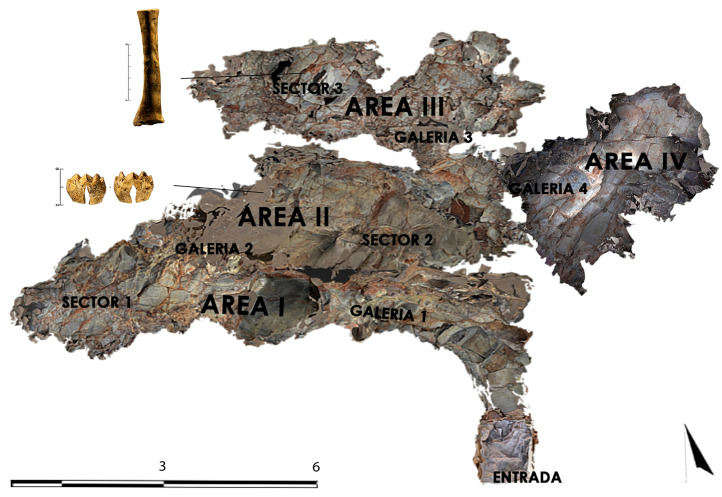
Map of the cave with the locations of the bone industry recovered from Gallery 2 (anthropomorphous) and Gallery 3 (eye idol). Picture modified from [19].

A partial anthropomorphic artefact was identified by the distal epiphysis of an unfused caprine metapodial that shows evidence of abrasion (TE18.BO.UE217.184). Maximum length: 18 mm, width: 23 mm, depth: 15 mm (location and picture in Figure 7).

**Gallery 3**

In Gallery 3, 672 bone remains were analyzed from the Bronze Age funerary context, five of which (0.74%) display cut marks (four slicing marks and one chop mark).

The locations of the cut marks are concentrated in the diaphysis of three long bones and one phalanx of a bovid (TE13.BO.UE126.861) interpreted as slicing marks. A chop mark was identified on the base of the left horncore of a caprine skull (TE17.BO.UE73.250) located in Inhumation 1 of Gallery 3. This chop mark suggests the removal of the horn from the skull or the removal of the horn sheath from the animal [44,51] (Figure 6A–C).

**Figure 8:**
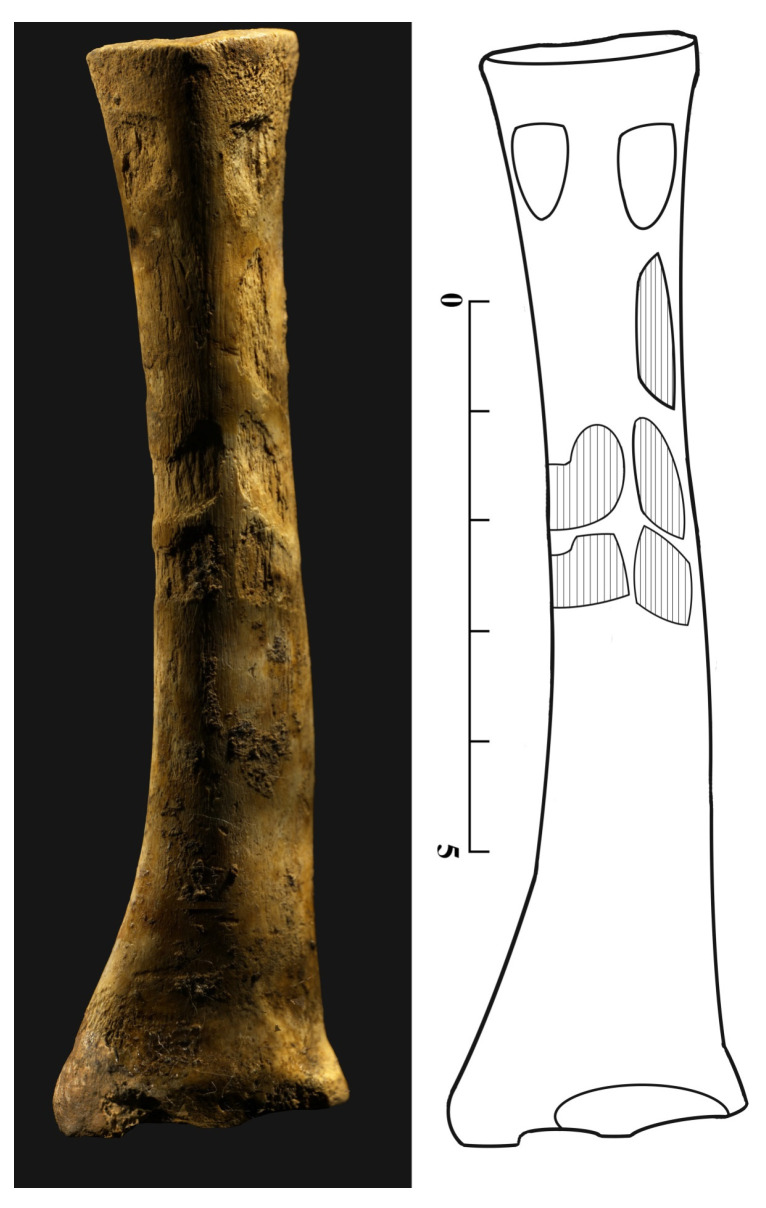
Picture of the eye idol and schematic drawing where incised eyes and decorations are observed.

A left radius of this caprine individual was recovered from Inhumation 4. Its distal epiphysis was cut transversally and polished after cutting. This object (TE17.BO.UE195.112) is what is known as an “eye idol”. From a dorsal view, incised eyes are observed, and a herringbone shape decoration was made by scraping. The radius dimensions (height: 95 mm, maximum width: 25 mm, thickness: 20 mm) attribute this to an adult caprine individual (Figure 7 for location and Figure 8).

**Gallery 4**

In the funerary context of Gallery 4, 1250 bone remains were identified.

Nine bone remains display cut marks. In SU 209, at least four slicing marks were found on a hyoid bone of one caprine (Figure 6D), and two slicing marks were observed on two rib fragments (TE18.BO.776; TE18.BO.1173). These slicing marks observed on these ribs and the caprine hyoid bone suggest filleting, which would have occurred in the process of removing the tongue from the head [52].

Evidence of fire modification is present on 20 bone remains (1.6%): 17 correspond to stage 3 (fully carbonized: completely black) and three correspond to stage 1 (less than half the surface is carbonized), according to [47]. Four bone remains display conspicuous tooth marks (pits or punctures) (209, 782, 1076, 1197), which were likely made by small carnivores. In SU224, rodent activity was documented on a caprine metatarsal (TE18.BO.691), and one long bone fragment was polished and altered by fire (partially black on the surface), which appears to be bone industry. In Gallery 4, 24 bone remains present biochemical alterations (dissolution but the causes are not clear)

## 4. Discussion

The faunal assemblage from the Castillejo de Bonete site is composed of domestic (*Ovis aries*, *Capra hircus*, *Bos taurus*, *Sus* sp. *Equus caballus*, and *Canis lupus familiaris*) and wild fauna (*Cervus elaphus*, *Capreolus capreolus*, *Oryctolagus cuniculus*, *Lepus granatensis* and *Meles meles*) [19,50,53]. Animals such as caprines, suids, canids, bovids, and equids have also been identified at the Motilla de Azuer, Motilla de Los Palacios [54,55], Motilla de El Retamar [55] and Motilla de Los Romeros [56], which is geographically close to the Castillejo del Bonete site.

In the tumular structure, the Great Tumulus 1 “deposits” contain few bone remains and are generally dental remains from domestic animals. The best represented taxa are the caprines. Such as in Tumulus 2, in Tomb 5, few bone remains were recovered (78:24, respectively), while sporadic (less than 1%) anthropic evidence (cut marks and fire alteration) on the bone remains was identified. In SU 194 (Tumulus 2), the almost complete caprine skeleton that was identified and the isolated bone remains of an immature suid were likely used as offerings. The scarcity of faunal bone remains in Tomb 5 prevents their clear interpretation.

In Enclosure 4, the high fracturation rate, together with the signs of fire damage on the bones, indicates possible animal consumption by humans. The biochemical modifications and marks (such as pits) indicate a long duration of exposure, during which the actions of small carnivores cannot be ruled out.

Most of the bone remains were found inside the cave. The caprines (probably *Ovis aries*, according to the results of the metrical analysis performed on the metacarpals) are the best represented taxa. Although the caprine skeletons were not found in anatomical connection, the associations of the anatomical elements forming complete or partial individuals, where elements of the skeleton are joined by anatomy and fusion stages, suggest that the animal carcasses were initially deposited as complete or almost complete in the different galleries. The complete and disarticulated anatomical elements of caprines that were recovered in every gallery and the refits of bone remains from different stratigraphical units (e.g., from Gallery 2) indicate the placement of the entire animals, their decomposition, disarticulation, and skeleton movement. This movement was most likely due to the repeated use of the cave.

In the stratigraphical units where the high caprine bone frequency allowed for the calculation of %MAU, we can observe an almost complete skeleton, and the anatomical parts with high flesh content (forelimbs, hind limbs) were well represented (75:40 in SU 217; 26.67: 33.33 in SU 26013: 18.75:26.67 in SU 209; 56.25: 63.33 in SU 210).

The age preference for sacrificing the caprines was for immature individuals. Most of these caprines were fetal, neonatal or juvenile individuals (63% in G2, 68.2% in G3, and 80% in G4).

The caprines have historically been an important animal in other Bronze Age sites belonging to “Motillas culture” in different contexts. In the Cerro de La Encantada (Ciudad Real) and Motilla de Santa Mª del Retamar (Argamasilla de Alba) sites, the caprines are the best represented group in the habitat context [55,57,58]. In the Bronze Age levels of the Motilla de Azuer site, caprines were always the first in importance [59].

Nevertheless, although it is located close to the “Motillas culture” sites, Castillejo del Bonete constitutes a monumental burial structure and astronomic building [14,15,19,60] similar to the Bocapucheros site (Campo de Calatrava), and while both are Bronze Age sites in La Mancha, they differ in terms of their solsticial character. This means that the Bocapucheros structure is oriented toward the Southern Cross. In contrast, Castillejo del Bonete is mainly oriented toward the sunrises and sunsets during the winter and summer solstices. This reflects different rituals in neighboring territories, which is possibly related to different dedications, though they are both astronomical. In the Bocapucheros site, *Ovis aries* dental remains were found in Tumulus 1 [61]. This suggests that the caprine is possibly a relevant and unique animal, as in Castillejo del Bonete.

Regarding the taphonomic aspects in the Castillejo del Bonete site, the almost complete absence of cut marks on the surfaces of the bones found there rules out the usual process undertaken during animal consumption. Nevertheless, some cut marks and fire modifications were observed (between 0 and 1% of the bone remains recovered). Two *Canis familiaris* remains depict cut marks, and thus dog consumption cannot be ruled out [53]. These sporadic anthropic modifications might indicate some type of commensalism ritual associated with the burial. The burning observed on the bones suggests the use of fire, which could also indicate some kind of ritual associated with the offering.

Two manufactured artifacts were identified: one eye idol (G3) and an anthromorph (G2), which are clearly symbolic.

In the case of the “eye idol”, the decoration consists of two recessed eyes under which at least three rows of herringbone motifs are etched, thought to be a representation of clothing, suggesting uniformity in the geometric design, which is an aspect that links it to the textile field [62]. This type of decoration on bone supports usually has irregular surfaces that stand out from the smooth natural surface.

The name “eye idols” on long bones is generalized from [63]. This term encompasses the carved decorations made on various bone supports, which both preserve the majority of the bone’s natural anatomy and whose length far exceeds their width. These idols are characteristic of the Iberian Peninsula, where there is a record preceding them in Neolithic ceramic decorations starting in the fifth millennium. For example, containers decorated with eyes have been found, showing that this theme was present in the Iberian Peninsula before the proliferation of this type of idol.

Most of these pieces are found in a funerary context, as is the case here, which suggests that they were created to give graphic support to the funerary ideology deployed. These objects, found in the tombs, may have acted as abstract representations of the deceased [64].

Another piece of bone industry was recovered from this site: an anthropomorphic artefact made of bone in SU 26013 (G2) [15,19].

The initial anatomical connection of the animals (especially the sheep), the selection of a very relevant species in this area (caprines), the high rate of skeletal representation, and the integrity of the anatomical elements with high content of meal, in addition to the preference for an age at sacrifice (mainly immature individuals), all suggest that the caprines were used as offerings, according to the criteria defined by [2]. All of this, together with the presence of the bone industry found on the anatomical elements of these caprines, undoubtedly suggests the likely special use of these animals, probably as offerings. Furthermore, the cave was not easily accessible for shepherds to bring their flocks inside; human bone remains were located in the cavity (funerary context), and there were almost no anthropic marks on the bone remains, which excludes other possible interpretations.

Feasting practices and the resulting meat offerings structured according to relatively standardized patterns were identified in Argaric sites (Bronze Age in the southeastern Iberian Peninsula) with a clear preference for the limbs of non-adult cattle, sheep and goats [65]. This is similar to Castillejo del Bonete, where the complete long bones are best represented and belong to non-adult caprines.

The meat offering of caprines in Argaric sites is found in tombs with lower quality grave goods and the burials of children under 12 as well as with adults of a lower social standing [65]. Future studies on the human remains and ornaments found close to the animals in the Castillejo del Bonete site will help to shed light on its inhabitants’ social structure and way of life.

The significant accumulation of microvertebrate bones showing signs of digestion to an extreme degree indicates consumption by small carnivores and suggests their occupation of the cave for extended periods [50]. This observation rules out a continuous human occupation of the cave throughout this time. During these periods, humans utilized the galleries primarily for funerary purposes. Caprines were offered as sacrifices, and evidence suggests that there were occasional rituals of shared meals or festivities as indicated by the sporadic consumption identified in some of the analyzed bones. This usage, while not consistent over time, was repeated due to the lack of anatomical connections among the almost complete skeleton elements. The reassembly of certain bones from different excavation units, along with the Bronze Age dates, suggests that both humans and small carnivores removed the bones that had been deposited in the cave.

## 5. Conclusions

Sheep have always played an important role in the societies occupying the southern Iberian Plateau. In the different contexts of the Castillejo del Bonete site, caprines (probably *Ovis aries*) are the best represented taxon. Domestic animals (caprines, *Bos taurus*, *Sus domesticus*, *Canis lupus familiaris*) and wild animals (*Cervus elaphus*, *Capreolus capreolus*, *Meles meles*, *Oryctolagus cuniculus* and *Lepus granatensis*) are also represented.

The faunal remains in the “deposits” of Great Tumulus 1, Tumulus 2, and Tomb 5 are interpreted as offerings represented as isolated bone, and the best represented taxa are the caprines.

In Enclosure 4, the anthropic modifications on the bone surfaces indicate the possible consumption of these animals.

In the Castillejo del Bonete cave, zooarchaeological and taphonomic analyses of faunal bone remains indicate that caprines were used as offerings in a funerary context, which was specifically associated with inhumations and within the pithoi. In geographical areas close to Castillejo del Bonete, such as the Argaric territories, animals such as cattle and caprines (sheep and goats) were found in burials interpreted as offerings and festive practices. The animal species accompanying human burials is associated with the deceased’s social status. In the case of Castillejo del Bonete, the study of the human remains associated with animals and their relationship with them will provide data and help to glean insight into the way of life and burials of this culture. The sporadic consumption of dogs, caprines, and bovines by humans cannot be ruled out in Castillejo del Bonete. This consumption may have been part of commensal activities or feasts related to funerals. The refitting of bone remains, as well as the associations between skeletons and their dating to the Bronze Age, suggest that the remains were moved within the cave over many years. Additionally, the cavity appears to have been used repeatedly by carnivores for an extended period, while human activity was more intermittent.

## Figures and Tables

**Table 1:** Radiocarbon dates from Castillejo del Bonete.

Lab. Code	Label/Location	Remains	Radiocarbon Age ^14^C BP	Cal BC (2 σ)	References
BETA-350768	Cave. Gallery 2, Survey W. TE12BO UE260 19	Human phalanx next to variscite	3870 ± 30	2465–2211	[12] p. 84.
POZ-67167	Cave. Gallery 2. Survey E. TE12BO UE26007	Branch of *Quercus ílex*. Bonfire on the floor at the foot of an anthropomorphic painting on the wall.	3385 ± 35	1769–1565	[16] p. 114.
ROME-1687	Great Tumulus 11. Tomb 4 (double inhumation)	Femur of Individual 2	3720 ± 70	2340–1921	[12] p. 84.
POZ-67168	Cave. Gallery 2. Survey E. TE12BO UE26015	*Rhamnus* sp. Sanitary cave cremation	3695 ± 35	2199–1977	[16] p. 114.
POZ-73665	Tumulus 2. Floor. TE13BO UE194	phalanx	3610 ± 35	2120–1885	[17]
PSUAMS-2077	Great Tumulus 1.Tomb1 TE03BO D8 UE12	Human dental piece	3565 ± 25	2014–1781	[49]
UCIAMS-205912	Cave Gallery 2 (SurveyW) TE12BO W UE26020	Mandible *Erinaceus europaeus*	3430 ± 20	1869–1665	[50]

**Table 2:** Metrical data from distal parts of *Ovis aries* metacarpals (DEM: medial trochlea depth; WCM: medial condyle width) from LASH, MNHN; *Capra hircus* from MNHN and Bronze Age caprines from the Castillejo del Bonete site.

	WCM	DEM
Castillejo del Bonete	10.03	9.7
	10	9.3
	10.3	9.1
	12.8	11.7
	10.8	11.37
*Ovis aries* (LASH)	14.58	12.76
	11.7	10.74
	9.89	10.21
	12.92	11.53
	11.5	10.3
	10.47	9.91
	10.4	9.9
	11.63	10.8
	13.08	11.63
	12.46	12.87
	10.69	9.14
	12.97	11.36
	12.92	11.77
	12.43	11.69
	13.31	11.94
	12.61	12.96
	14.46	12.88
	13.7	11.82
*Ovis aries* (MNHN)	12.71	11.02
	14.12	13.14
	11.74	12.69
	15.73	12.4
	12.53	12.86
	12.38	12.07
*Capra hircus* (MNHN)	13.62	9.11
	13.06	10.57
	12.9	8.31
	13.78	11.99
	13.41	10.73
	10.44	8.76
	11.93	10.12

**Table 3:** NISP from Gallery 2 by stratigraphical units. C: caprines; *Oa*: *Ovis aries*; *Bt*: *Bos taurus*; L indet.: Leporidae indet.; *O cun*: *Oryctolagus cuniculus*; *Ce*: *Cervus elaphus*; *Cf*: *Canis familiaris*.

Gallery2/ NISP	207	212	217	218	228	229	230	26006	26010	26013	26014	26015	26019	26025	26028	26029
Caprines	5	2	39	3	36	29	4	1	1	56					3	1
*Ovis aries*										1						
*Bos taurus*					8	1				1						
*Sus* sp.			2		1	9				5				1		
*Equus caballus*										1						
Leporidae indet.		3	6	1						22	1					
*Ocun*	6				1	24				11					2	
*Lepus granatensis*		1	5	1		5				21						
*Cervus elaphus*	1		2			2	3			3						1
Carnivora indet.						6				4	2			1		
*Canis familiaris*	1									1						
*Meles meles*			4	1		6				7						
Birds						1	1									
LS			2	1	4	5	2			5						
MS	2	4	14	2	7	67	3	9	2	97		1	3			1
SS	7		3	1	1	4		1		16						
TOT	22	10	77	10	58	159	13	11	3	251	3	1	3	2	5	3

**Table 4:** MNI of caprines with age at death by stratigraphical units from Gallery 2. F: fetal; N: neonatal; J: juvenile; SA: subadult; A: adult; C: complete skeleton; P: partial skeleton; isol: isolated anatomical elements.

**Gallery 2**	**SU**	**F**	**N**	**J**	**SA**	**A**	**S**	**Indet.**	**Total**
	207			1 (P)					1
	212							1	1
	217	1 (P)	1 (C)						2
	228			1 (P)		1 (P)			2
	229			1 (P)		1 (P)			2
	230			1 (isol)		1 (isol)			2
	26006							1	1
	26010							1	1
	26013	1 (P)				1 (P)			2
	26028							1	1
	26029							1	1
	Total	2	1	4	0	4		5	16

**Table 5:** NISP from Gallery 3 by stratigraphical units. C: caprines; *Oa*: *Ovis aries*; *Bt*: *Bos taurus*; L indet.: Leporidae indet.; *O cun*: *Oryctolagus cuniculus*; *Ce*: *Cervus elaphus*; *Cf*: *Canis familiaris*.

**Gal3/NISP**	**41 (s.3.1)**	**42 (s.3.1)**	**49**	**59 (s.3.1)**	**73 (s.3.1)**	**78 (s.3.1)**	**91 (s.3.0)**	**93**	**94**	**99**	**114 (s.3.1)**	**116 (s.3.0)**	**126 (s.3.0)**	**127 (s.3.0.4**	**199**	**200 (s.3.0)**	**127 (s.3.0.4**	**129 (s.3.1)**
Caprines	1	4	10		11	2	10	2		1	14	1	3	16	5	13	16	2
*Oar*													1					
*Bt*					1						2		2			3		1
*Sus* sp.					1				3		5	3	3	4		4	4	
Lep ind.					2								2	10			10	
*O cun*			5	1	1						1		3			8		
*Lepus* sp.		2					1				1		2	2			2	
*Cer elap*		1											1			4		
*Capr capr*																		
Carn indet.														1			1	
*Canis fam*																		
*Mm*																		1
Avif													5					
TG								1						1		3	1	
TM		6	8	20	9	10	5				3	3	13	16	1	21	16	5
TP		1		1									1			30		
TOT	1	14	23	22	25	12	16	3	3	1	26	7	36	50	6	86	50	9
**Gal3/NISP**	**134 (s.3.0)**	**140 (s.3.1)**	**141 (s.3.0)**	**143 (s.3.1)**	**158**	**160 (s.3.1.**	**162 (s.3.0)**	**166**	**182 (s.3.2)**	**185 (s.3.3)**	**191 (s.3.4)**	**192 (s.3.0)**	**193**	**195 (s.3.0)**	**199**	**200 (s.3.0)**		
Caprines		13	3	1	3	2	3	13	48	6	7	1		6	5	13		
*Oar*			1															
*Bt*					4			2	2							3		
*Sus* sp.					1				8	3	1					4		
Lep ind.	2	1	2						1		6			1				
*O cun*	1					1			6	1				1		8		
*Lepus* sp.	1								5	1	1			1				
*Cer elap*	2								7					2		4		
*Capr capr*									1									
Carn indet.	1																	
*Canis fam*		1							1									
*Mm*			1	2														
Avif																		
TG	2		2		1			1	12							3		
TM	6	18	4	11	5	6		8	53	8	5	2	2	2	1	21		
TP									3	3						30		
TOT	15	33	13	14	14	9	3	24	147	22	20	3	2	13	6	86		

**Table 6:** MNI of caprines with the age at death by stratigraphical units from Gallery 3. F: fetal; N: neonatal; J: juvenile; SA: subadult; A: adult; C: complete skeleton; P: partial skeleton.

**Gallery 3**	**SU/Age Category**	**F**	**N**	**J**	**SA**	**A**	**S**	**Indet.**	**Total**
	41							1	1
	42							1	1
	49							1	1
	73				1 (P)				1
	78		1 (P)						1
	91		1 (P)			2 (P)			3
	93			1 (P)					1
	114		1 (P)			1 (P)			2
	116							1	1
	126							1	1
	127	1 (P)		1(P)		1 (P)			3
	129							1	1
	134							1	1
	140							1	1
	141								1
	143								1
	158				1 (P)				1
	162							1	1
	166				2 (P)				2
	182	2 (P)	1 (P)			1 (P)			4
	185				1 (P)				1
	191				1 (P)				1
	195					1 (P)			1
	199							1	1
	200					1 (P)			1
	TOTAL	3	4	2	6	7		10	32

**Table 7:** NISP from Gallery 4 by stratigraphical unit.

Gallery 4/NISP	156	202	206 (pit 2)	209	210	211	212	224	225	244 (pit 1)	309
Caprines	16	16	21	87	232	2	1	4	15	5	9
*Bos taurus*	1		1	1	26					2	
*Sus* sp.	2	3		2	18				1	1	
*Equus caballus*					1						
Leporidae indet.			5		3				2		
*Oryctolagus cuniculus*	7	2		8	20				2	2	
*Lepus* sp.	6	1	1						1	1	
*Cervus elaphus*	2	3			1						
Carnivora indet.					6						
*Canis familiaris*			1		3				2		
*Meles meles*	2										
LS				4	1				2		
MS	46	6	8	32	310				16		
SS	3	4		1	23				1		
TOTAL	86	37	37	135	627	2	1	4	42	11	9

**Table 8:** MNI of caprines with the age at death by stratigraphical units from Gallery 2. F: fetal; N: neonatal; J: juvenile; SA: subadult; A: adult; C: complete skeleton; P: partial skeleton, isol: isolated anatomical elements.

**Gallery 4**	**SU/Age Category**	**F**	**N**	**J**	**SA**	**A**	**S**	**Indet.**	**MNI**
	156			1 (P)		1 (P)			2
	202		1 (P)			1 (P)			2
	206	1 (P)	1 (P)	1 (P)					3
	209		1 (C) + 2 (P)			1 (P)			4
	211							1	1
	224	1 (isol)		1 (isol)					2
	225			1 (P)					1
	244			1 (P)					1
	Total	2	5	5	0	3	0	1	16

## Data Availability

Supplementary Information (SI1) is included for detailed data.

## Supplementary Materials

The following supporting information can be downloaded at https://www.mdpi.com/article/10.3390/ani15050680/s1.
