# Supplementary material for: Animals for the Deceased: Zooarchaeological Analysis of the Bronze Age in the Castillejo del Bonete Site (Terrinches, Ciudad Real, Spain)
Source: Animals (Basel). 2025 Feb 26;15(5):680. doi: 10.3390/ani15050680 (PMC11899635; doi:10.3390/ani15050680)
Supplement: Supplementary file 1 [file animals-15-00680-s001.zip › animals-3431970-supplementary.pdf]

SUPLEMENTARY INFORMATION

Gallery 2.

-In stratigraphical unit 217, the best represented taxa are the caprines with 39 bone remains identified, belonging to a minimum of two individuals (one partial fetus and one almost complete neonate): Leporidae indet. (6), *Lepus granatensis* (5), *Meles meles* (4), *Sus* sp. (2), *Cervus elaphus* (2), large sized animals (2), medium sized animals (14), and small sized animals (3).

- In SU 229, a total of 159 bone faunal remains have been identified: 29 belong to a minimum number of two caprines (one juvenile and one adult), represented by a partial skeleton (Table 4), *Oryctolagus cuniculus* (24), *Sus* sp. (9), Carnivora indet. (6), *Meles meles* (6), *Lepus granatensis* (5), *Bos taurus* (1), bird (1), large sized animals (5), medium sized animals (67). A metatarsal diaphysis fragment from a caprine appears to be bone industry (TE18.UE229.221).

-In SU 26013, a total of 251 faunal bone remains have been identified. The best represented group are the caprines (57) with a minimum number of two individuals (one fetal and one adult) represented by partial skeletons: Leporidae indet. (22), *Lepus granatensis* (21), *Oryctolagus cuniculus* (11), *Meles meles* (7), *Sus* sp. (5), Carnivora indet. (4), *Bos taurus* (1), *Canis familiaris* (1), *Equus caballus* (1), large sized animals (5), medium sized animals (97), and small sized animals (16).

Gallery 3

Sector 3.0. includes:

Sector 3.0.0.is constituted by SU 91, 116, 162, 192, 200.

In SU 200, a number of the 86 bone remains have been identified: 13 belong to caprines, which are represented by the partial skeleton of one individual, *Sus* sp. (4: MNI=2: one immature and the other is female, determined by the canine), *Cervus elaphus* (4), *Bos taurus* (4), *Oryctolagus cuniculus* (8), large animals (3), medium animals (21), and small animals (30).

Sector 3.0.1. is constituted by SU 134 and 141.

Sector 3.0.2. is constituted by SU 94 and 99. In SU 94, three dental remains have been identified: two middle mandibles with dp_3, dp_4 belonging to an immature individual of *Sus* sp. and one incisive belonging to an adult suid. In SU 99, there is a third lower molar that has been identified as belonging to one adult caprine.

Sector 3.0.3. **Inhumation 4** was defined and includes the following:

SU 126. 36 faunal bone remains were recovered, four of which belong to an adult ovicaprine. These bone remains belong to *Ovis aries,* according to metrical data taken from a distal metacarpal (Fig. 3).

SU 195. Human bone remains were found and 13 faunal bone remains were discovered closeby, six of which belong to an adult caprine.

Sector 3.0.4. **Inhumation 5** was recovered from this sector, which is constituted by SU 127. 50 bone faunal remains have been identified: 16 bone remains belong to a minimum number of three caprine individuals (one fetal, one juvenile, and one adult), which are represented by partial skeletons: Leporidae indet. (10), *Sus* sp. (4), *Lepus granatensis* (2), Carnivore indet. (1), large sized animal (1), and medium sized animals (16).

Sector 3.0.6. includes SU 140.

Sector 3.0.9. includes SU 162.

Sector 3.1. includes:

Inhumation 1:

This inhumation is constituted by stratigraphical unit 61 and SU 73 (s.3.1.7)

In SU 61, three faunal bone remains have been identified: one caprine and two medium sized animals.

In SU 73, a total of 25 bone remains have been identified: 11 of which belong to a subadult caprine individual represented by a partial skeleton.

Inhumation 2 (3.1.7) is constituted by stratigraphical unit 160.

This inhumation includes SU 160, where nine faunal bone remains have been identified: caprines (2), *Oryctolagus cuniculus* (1), and medium sized animals (6).

Inhumation 3 is located in the center of sector 3.1. and is constituted by stratigraphical unit 145. Faunal bone remains were not found close to the human bone remains.

Sector 3.1 is completely separated from sectors 3.2, 3.3. and 3.4.

Sector 3.2 includes SU49 (23 bone remains) and SU 182:

In SU 182, a total of 147 bone faunal remains have been identified: 48 belong to a minimum of four caprines represented by partial skeletons (two fetals, one neonatus and one adult), *Sus* sp. (8), *Cervus elaphus* (7), *Oryctolagus cuniculus* (6), *Lepus granatensis* (5), *Bos taurus* (2), *Capreolus capreolus* (1), *Canis familiaris* (1), large sized animals (12), medium sized animals (53), and small sized animals (3).

Gallery 4

SU 156

A total of 86 bone remains have been identified in SU156: caprines (NISP=16: MNI=2; one juvenile and one adult), *Bos taurus* (1 phalanx fsing: MNI=1), *Sus* sp. (2:1), lagomorphs (13: 6 *Lepus* sp. and 7 *Oryctolagus cuniculus*), Carnivora indet. (2); avian faunal remains (one complete skull), *Meles meles* (2:1), 46 bone fragments from medium sized animals, and three bone remains from small sized animals.

One point of a horncore from one ovicaprine is polished and appears to be bone industry.

SU 202

A total of 37 faunal bone remains have been recovered from SU 202: caprines (16:2). These belong to a minimum of two individuals: one less than three months of age (unfused distal femur: neonate), and another one was adult (with permanent molars): *Sus* sp. (3:1), *Oryctolagus cuniculus* (2:1), *Lepus* sp. (1:1), *Cervus elaphus* (3:1), medium sized animals (8), and small sized animals (4).

Sector 4.1.

SU 225

42 faunal bone remains have been recovered. The best represented taxa are caprines (15:1), *Sus* sp. (1:1), *Oryctolagus cuniculus* (2:1), *Lepus* sp. (1:1), and Leporidae indet. (2), *Canis familiaris* (2:1), medium sized animals (16), big sized animals (2), and small sized animals (1).

Sector 4.3

SU 206.

This stratigraphical unit is defined as pithos 2, where human remains were found and 37 bone faunal remains were identified close to them. Caprines are the best represented taxa (21: 3) with a minimum of three individuals (one fetal; one neonatus and one juvenile, partially represented by the carcasses); *Bos taurus* (1:1), Leporidae indet. (5:1), *Lepus granatensis* (1:1), *Canis familiaris* (1:1), and medium sized animals (8).

The three caprine individuals are represented in the partial category with the majority of the anatomical elements complete and not broken
